# The prognostic value of multiparametric cardiac magnetic resonance in patients with systemic light chain amyloidosis

**DOI:** 10.3389/fonc.2023.1069788

**Published:** 2023-05-03

**Authors:** Fujia Miao, Chunxiang Tang, Guisheng Ren, Jinzhou Guo, Liang Zhao, Weiwei Xu, Xiyang Zhou, Longjiang Zhang, Xianghua Huang

**Affiliations:** ^1^National Clinical Research Center of Kidney Diseases, Affiliated Jinling Hospital, Medical School of Nanjing University, Nanjing, China; ^2^Department of Radiology, Affiliated Jinling Hospital, Medical School of Nanjing University, Nanjing, China

**Keywords:** light chain amyloidosis, cardiac magnetic resonance imaging, late gadolinium enhancement, T1 mapping, prognosis

## Abstract

**Background:**

Late gadolinium enhancement (LGE) is a classic imaging modality derived from cardiac magnetic resonance (CMR), which is commonly used to describe cardiac tissue characterization. T1 mapping with extracellular volume (ECV) and native T1 are novel quantitative parameters. The prognostic value of multiparametric CMR in patients with light chain (AL) amyloidosis remains to be thoroughly investigated.

**Methods:**

A total of 89 subjects with AL amyloidosis were enrolled from April 2016 to January 2021, and all of them underwent CMR on a 3.0 T scanner. The clinical outcome and therapeutic effect were observed. Cox regression was used to investigate the effect of multiple CMR parameters on outcomes in this population.

**Results:**

LGE extent, native T1 and ECV correlated well with cardiac biomarkers. During a median follow-up of 40 months, 21 patients died. ECV (hazard ratio [HR]: 2.087 for per 10% increase, 95% confidence interval [CI]: 1.379-3.157, P < 0.001) and native T1 (HR: 2.443 for per 100 ms increase, 95% CI: 1.381-4.321, P=0.002) were independently predictive of mortality. A novel prognostic staging system based on median native T1 (1344 ms) and ECV (40%) was similar to Mayo 2004 Stage, and the 5-year estimated overall survival rates in Stage I, II, and III were 95%, 80%, and 53%, respectively. In patients with ECV > 40%, receiving autologous stem cell transplantation had higher cardiac and renal response rates than conventional chemotherapy.

**Conclusion:**

Both native T1 and ECV independently predict mortality in patients with AL amyloidosis. Receiving autologous stem cell transplantation is effective and significantly improves the clinical outcomes in patients with ECV > 40%.

## Introduction

Systemic light chain (AL) amyloidosis is a relatively rare hematological disorder characterized by the deposition of misfolded monoclonal immunoglobulin light chains in the form of amyloid fibrils. Insoluble fibrils accumulate extracellularly, and gradually affect the structure and function of the involved tissues and organs (1, 2). Cardiac involvement is frequent and portends the prognosis of patients with AL amyloidosis (3). Therefore, early identification of cardiac involvement and risk stratification are important for these patients to improve clinical outcomes. Currently, the Mayo staging system based on cardiac biomarkers is widely used in clinical practice (4, 5). However, the concentration of serum cardiac biomarkers, including amino-terminal pro-brain natriuretic peptide (NT-proBNP) and troponin-T (TnT) or troponin-I, may be confounded by renal function or other cardiovascular diseases. Additionally, these biomarkers depend on measuring surrogates rather than directly measuring myocardial interstitial dilation.

Cardiac magnetic resonance (CMR) is a robust technique to provide morphological and functional parameters and other novel quantitative parameters. Late gadolinium enhancement (LGE) is a classic noninvasive protocol for visualizing myocardial tissue characterization, which reflects the expansion of myocardial interstitium. Circumferential subendocardial LGE has been reported to be a typical finding in AL amyloidosis, and a more diffuse transmural LGE pattern has been associated with transthyretin amyloidosis (6, 7). A typical LGE pattern can not only diagnose cardiac amyloidosis with high sensitivity and specificity, but also serves as a prognostic factor (8–10). Despite the diagnostic and prognostic values of LGE pattern, cardiac amyloidosis may not have a typical LGE pattern or may present as patchy LGE in early stages of disease. Native T1 and extracellular volume (ECV), as a marker of infiltration, have been shown to correlate with amyloid burden and have excellent diagnostic accuracy (11, 12). Furthermore, Native T1 and ECV had the potential to become a predictor of mortality in few studies (13, 14). However, the parameters that have an independent predictive effect on mortality need to be selected from multiparametric CMR, and the prognostic value of left ventricle LGE extent remains to be investigated.

The aim of this study was to investigate the prognostic value of CMR parameters in a cohort of AL patients and to observe treatment-related response in these patients.

## Materials and methods

### Study population

**Figure 1:**
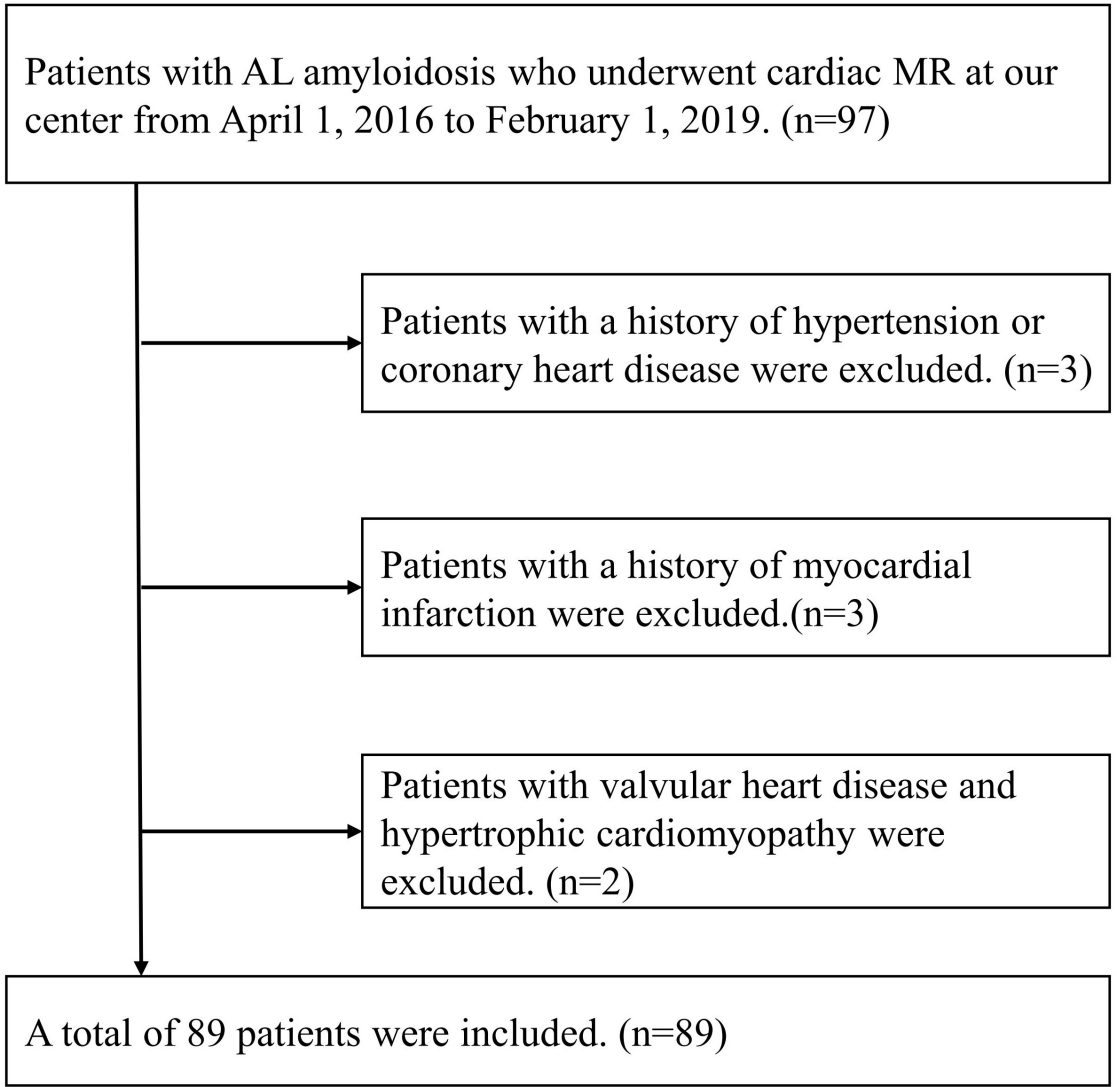
Inclusion and exclusion criteria of this study.

From April 1, 2016 to January 30, 2019, ninety-seven consecutive AL amyloidosis patients without contraindications to CMR were recruited at our institution. Patients with estimated glomerular filtration rate > 30 ml/min/1.73m^2^ were eligible for LGE imaging. The inclusion criteria were patients with histologically confirmed AL amyloidosis by Congo red and immunofluorescence staining. Of the 97 patients with AL amyloidosis enrolled, 8 patients were excluded due to with a history of hypertension or coronary heart disease (n=3), a history of myocardial infarction (n=3) and valvular heart disease or hypertrophic cardiomyopathy (n=2). Eventually, 89 patients with AL amyloidosis were included in our study (**Figure 1**). This study was carried out with the approval of the ethical review committee of our hospital and in accordance with the Declaration of Helsinki.

### Relative definitions

The definition of organ involvement was based on the criteria proposed at the 10^th^ International Symposium on Amyloid and Amyloidosis (15). Clinical diagnostic criteria for cardiac amyloidosis (CA) included either of the followings (1): interventricular septal thickness > 12 mm in the absence of other cardiac diseases or (2) NT-proBNP > 332 pg/ml in the absence of renal insufficiency or atrial fibrillation. The criteria of hematological response was defined according to the standard established by the International Society of Amyloidosis (16). Cardiac and renal responses were defined according to previous literature (17, 18). Hematological, cardiac and renal responses were evaluated every 1 to 3 months within the first year after treatment and every 6 to 12 months thereafter. The best hematological and organ responses were used in this study.

### CMR protocols and image analysis

Standard CMR was performed using a 3.0 T scanner (TIM Trio, Siemens, Germany). The CMR protocols included cine imaging, LGE imaging and T1 mapping. Cine images were available in short-axis view, two- and four-chamber long-axis views during end-expiratory breath-hold *via* a steady-state free precession sequence. LGE imaging was performed using an electrocardiograph-triggered phase-sensitive inversion recovery (PSIR) sequence, and the images were collected 5-8 minutes after the gadolinium injection. Native and post-contrast T1 were obtained by a modified Look-Locker Inversion recovery (MOLLI) sequence.

All images were analyzed through a semi-automatic postprocessing software CVI42 (version 5.3, Circle Cardiovascular imaging, Calgary, Canada). The structural and functional parameters of the heart were obtained by analyzing short axis cine images at the end of systolic and diastolic phases. For T1 mapping and LGE quantification, an experienced cardiovascular radiologist manually outlined the endocardial and epicardial contours of left ventricle in short axis images. A user-defined referral area was drawn in normal myocardium, and regions of LGE were defined when the signal intensity exceeded 5 standard deviations (SD) of the reference area. The region of interest for T1 mapping and ECV measurements was left ventricular myocardium. Native T1 and ECV were quantitatively measured in the 16 American Heart Association (AHA) segments of the left ventricle, and global left ventricular native T1 and ECV were used for further analysis. The software calculated the LGE volume of each slice of the left ventricle in short axis images, defined as the sum of the LGE area of each slice multiplied by the distance between the two slices. LGE extent was then expressed as the sum of LGE volumes of each slice divided by the total myocardial volume of the left ventricle.

### Follow-up

The end-point of this research was all-cause mortality. Overall survival (OS) was calculated from the date of performing CMR until death due to any cause. All patients received appropriate treatment after diagnosis. Follow-up was performed by a clinician blinded to the results of CMR parameters and clinical status. For patients who were lost to follow-up, data from their last clinical visit were used for further analysis. The deadline of follow-up was August 1, 2021.

### Inter-observer variability

Inter-observer variability for ECV, native T1 and LGE extent was independently evaluated by two experienced cardiovascular radiologists in twenty-five randomly selected patients.

### Statistical analysis

Statistical analysis was performed with SPSS software (Version 23, IBM, Armonk, New York) and MedCalc software (version 20.0). Normally distributed continuous variables were presented as mean ± SD, and comparisons between groups were performed using Student ‘s t test. Continuous variables that were not normally distributed were presented as median (interquartile range), and compared between groups using the Mann-Whitney U-test. The differences between categorical data were compared using chi-square test or Fisher’s exact test. Correlations between parameters were evaluated using Spearman’s rho correlation analysis. Spearman’s rho coefficients were presented in terms of rs. Inter-class correlation coefficients (ICC) were performed to detect variations in the ECV, native T1, and LGE extent between observers.

Survival was assessed by Kaplan–Meier curves and Cox proportional hazards regression analysis. The median values of ECV and native T1 were used as cut-off values in Kaplan–Meier curves. A two-tailed p value of < 0.05 was considered statistically significant.

## Results

### Demographic characteristics

The demographic and clinical features of the cohort are displayed in **Table 1**, and the baseline CMR parameters are summarized in **Table 2**. Patients in this study were classified into CA or non-CA according to the definition of cardiac involvement. Compared with non-CA patients, patients with CA had significantly decreased systolic blood pressure (P=0.008), increased the difference between involved and uninvolved free light chains (dFLC) (P=0.012), involved free light chains (P=0.017) and bone marrow plasma cells (P=0.008). In terms of cardiac biomarkers, patients with CA had significantly increased NT-proBNP (P < 0.001) and TnT (P < 0.001). There were significant differences in CMR parameters between the two groups, except for left ventricle end-diastolic volume index (LVEDVI), left ventricle end-systolic volume index (LVESVI), stroke volume index (SVI), cardiac index (CI) and left ventricular ejection fraction (LVEF).

**Table 1:** Clinical baseline characteristics of the cohort.

	All patients (n=89)	CA (n=55)	Non-CA (n=34)	P value
Age, years	53.0 ± 7.6	52.8 ± 7.9	53.3 ± 7.2	0.748
Gender, male/female, n (%)	49 (55)/40 (45)	27 (49)/28 (51)	22 (65)/12 (35)	0.150
Systolic blood pressure, mmHg	116 ± 15	113 ± 14	122 ± 16	0.008
Lambda restricted, n (%)	77 (86.5)	49 (89.1)	28 (82.4)	0.559
Involved organ, n (%)				
Kidney	89 (100)	55 (100)	34 (100)	–
Liver	4 (4.5)	3 (5.5)	1 (2.9)	0.976
Nerve	9 (10.1)	5 (9.1)	4 (11.8)	0.964
Laboratory date				
Urinary proteinuria, g/24h	3.8 (2.0-6.1)	4.6 (2.3-9.1)	3.4 (1.6-5.2)	0.071
Serum albumin, g/L	28.3 (24.2-33.4)	27.8 (24.3-33.2)	28.4 (24.0-34.4)	0.748
Serum creatinine, mg/dl	0.81 (0.69-1.01)	0.82 (0.69-1.03)	0.79 (0.68-0.99)	0.645
dFLC, mg/L	76.7 (26.2-142.3)	84.8 (50.3-143.2)	44.4 (12.5-131.2)	0.012
iFLC, mg/L	89.8 (43.8-153.7)	102.7 (69.1-160.0)	65.6 (26.2-139.3)	0.017
BMPCs, %	2.5 (1.0-5.3)	3.0 (2.0-6.5)	2.0 (1.0-3.1)	0.008
Cardiac biomarkers				
NT-proBNP, pg/ml	674 (103-2403)	2121 (748-3462)	81 (50-119)	< 0.001
Troponin T, ng/ml	0.027 (0.011-0.051)	0.043 (0.027-0.078)	0.010 (0.005-0.015)	< 0.001
First-line therapy				
ASCT, n (%)	63 (70.8)	34 (61.8)	29 (85.3)	0.018
Bortezomib, n (%)	17 (19.1)	13 (23.6)	4 (11.8)	0.166
IMiDs, n (%)	7 (7.9)	7 (12.7)	0 (0)	0.041
Others, n (%)	2 (2.2)	1 (1.8)	1 (2.9)	1.000

CA, cardiac amyloidosis; dFLC, difference between involved and uninvolved free light chains; iFLC, involved free light chains; BMPCs, bone marrow plasma cells; NT-proBNP, N-terminal pro-B-type natriuretic peptide; ASCT, autologous stem cell transplantation; IMiDs, immunomodulatory drugs.

**Table 2:** Baseline characteristics based on CMR of the cohort.

	All patients (n=89)	CA (n=55)	Non-CA (n=34)	P value
LVEDVI, ml/m²	57.5 (48.3-64.9)	57.9 (46.9-63.8)	54.4 (50.2-65.6)	0.717
LVESVI, ml/m²	20.5 (15.3-28.5)	20.5 (14.7-31.3)	20.9 (15.9-26.4)	0.873
SVI, ml/m²	33.1 (29.0-23.2)	32.9 (25.5-38.6)	35.3 (29.8-40.6)	0.066
LVMI, g/m^2^	62.1 (51.1-82.8)	74.7 (53.9-94.9)	55.6 (46.1-63.8)	< 0.001
LV GPWT (Diast), mm	13.4 (11.3-16.0)	15.0 (12.8-18.2)	11.6 (10.6-13.4)	< 0.001
CI, L/min/m²	2.74 (2.24-3.13)	2.73 (2.21-3.10)	2.82 (2.25-3.17)	0.698
LVEF, %	63.4 (53.6-70.8)	64.3 (50.2-70.5)	63.0 (57.4-71.2)	0.432
Native T1, ms	1344 (1280-1449)	1423 (1344-1484)	1273 (1240-1314)	< 0.001
ECV, %	40.4 (33.3-52.0)	49.4 (40.4-55.7)	31.7 (28.6-35.3)	< 0.001
LGE extent, %	19.6 (4.5-57.5)	49.0 (15.1-65.4)	4.8 (1.6-16.2)	< 0.001

CA, cardiac amyloidosis; LVEDVI, left ventricle end-diastolic volume index; LVESVI, left ventricle end-systolic volume index; SVI, stroke volume index; LVMI, left ventricle mass index; LV GPWT (Diast), left ventricle global peak wall thickness (Diastolic); CI, cardiac index; LVEF, left ventricular ejection fraction; ECV, extracellular volume; LGE, late gadolinium enhancement. LGE extent, native T1 and ECV were quantitative parameters for the left ventricle.

In this cohort, sixty-three (71%) patients had received ASCT, of whom four patients did not receive induction chemotherapy prior to transplantation and fifty-nine patients received induction chemotherapy. Twenty-four (27%) patients received chemotherapy as a first-line regimen that included bortezomib or immunomodulatory drugs (IMiDs). Two (2%) patients just received support treatment due to poor general conditions. Eighty (90%) patients did not receive any treatment at the time of CMR scanning. Nine (10%) patients underwent CMR scanning after treatment, including seven (78%) who received bortezomib-based induction therapy prior to transplantation and two (22%) who received IMiDs before CMR scanning.

### Hematological and organ responses

The hematological and organ responses of patients are shown in **Table 3**. 18 patients had a baseline dFLC < 20 mg/L, and 3 patients lacked a clinical follow-up date, so a total of 68 patients could assess hematological responses. In all patients, the hematological overall response rate, cardiac and renal response rate was 83.8%, 65.4% and 67.5%, respectively. There were 45 patients with ECV > 40%, and these patients were divided into an ASCT group and a chemotherapy group according to the treatment regimens. The ASCT group had a higher rate of cardiac response (77.3% *vs* 35.3%, P=0.008) and renal response (75.0% *vs* 31.3%, P=0.006) than the chemotherapy group. Baseline characteristics of patients receiving ASCT and chemotherapy are shown in **Supplementary Table 1.**

**Table 3:** The hematological response and organ response in all patients and subgroups.

	All patients N=89	ECV > 40% N=45	ASCT (ECV > 40%) N=24	Chemotherapy (ECV > 40%) N=21	P value
Hematological response					
ORR % (n)	83.8 (57/68)	78.4 (29/37)	90.5 (19/21)	62.5 (10/16)	0.055
CR % (n)	50.0 (34/68)	40.5 (15/37)	47.6 (10/21)	31.3 (5/16)	0.315
VGPR % (n)	25.0 (17/68)	27.0 (10/37)	33.3 (7/21)	18.8 (3/16)	0.538
PR % (n)	8.8 (6/68)	10.8 (4/37)	9.5 (2/21)	12.5 (2/16)	1.000
NR % (n)	16.2 (11/68)	21.6 (8/37)	9.5 (2/21)	37.5 (6/16)	0.055
Organ response					
Cardiac % (n)	65.4 (34/52)	59.0 (23/39)	77.3 (17/22)	35.3 (6/17)	0.008
Renal % (n)	67.5 (54/80)	57.5 (23/40)	75.0 (18/24)	31.3 (5/16)	0.006

ASCT, autologous stem cell transplantation; ORR, overall response rate; CR, complete response; VGPR, very good partial response; PR, partial response; NR, no response

### Reproducibility of LGE extent, native T1 and ECV measurements

The ICC values in inter-observer reproducibility for LGE extent, native T1 and ECV were 0.870 (95% CI: 0.730-0.940, P < 0.001), 0.964 (95% CI: 0.921-0.984, P < 0.001) and 0.967 (95% CI: 0.928-0.985, P < 0.001), respectively.

### Correlation between ECV, native T1, LGE extent and other parameters

**Figure 2:**
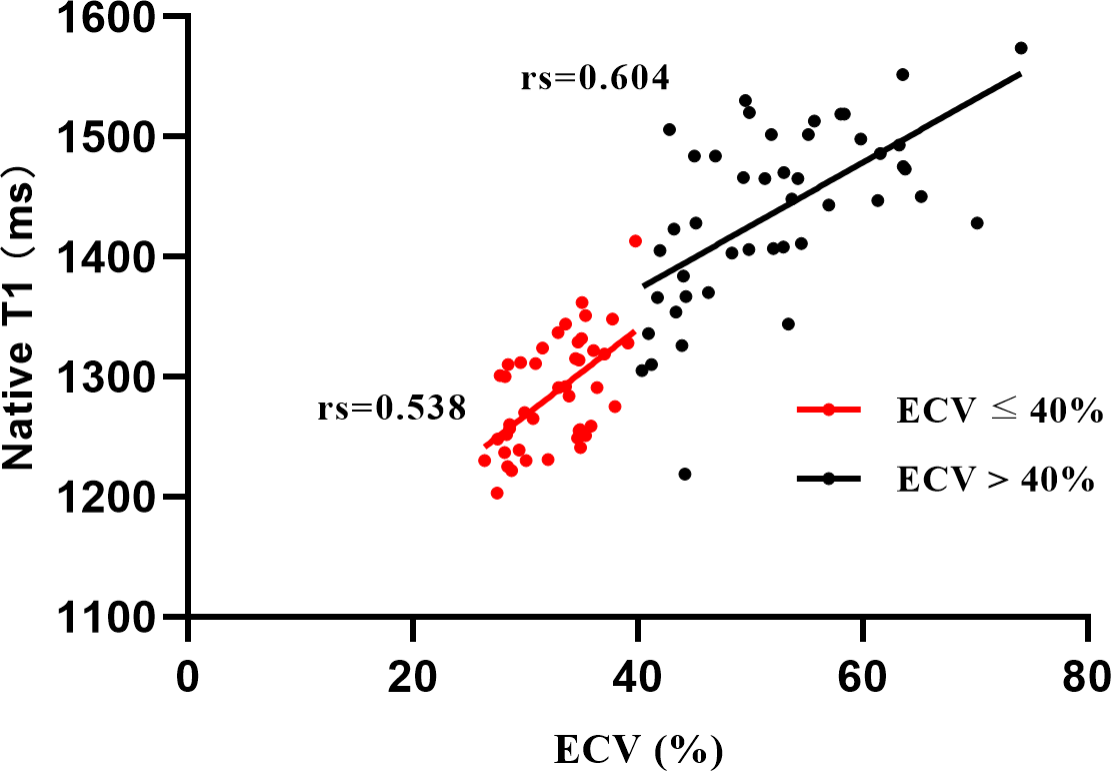
Correlation among native T1 and extracellular volume (ECV) in groups depending on ECV values, ECV ≤ 40% (red), and ECV > 40% (black).

As shown in **Table 4**, ECV, native T1 and LGE extent correlated well with clinical indicators and CMR parameters. ECV and native T1, ECV and LGE extent showed significant correlations with each other (rs=0.850, rs=0.815, respectively). Furthermore, in the two groups with ECV > 40% or ECV ≤ 40%, the correlation between ECV and native T1 was similar (rs=0.604, rs=0.538, respectively) (**Figure 2**). We also found that the correlation between Mayo 2012 Stage and ECV was better than that between Mayo 2012 Stage and native T1 or LGE extent.

**Table 4:** Native T1, ECV and LGE extent correlation with clinical indicators and other cardiac MR parameters in AL patients.

	ECV	Native T1	LGE extent
Mayo 2004 Stage	0.538*	0.556*	0.355
Mayo 2012 Stage	0.662*	0.607*	0.452*
NT-proBNP, pg/ml	0.783*	0.737*	0.562*
TnT, ng/ml	0.725*	0.669*	0.609*
LVMI, g/m^2^	0.650*	0.616*	0.616*
LV GPWT (Diast), mm	0.685*	0.656*	0.590*
ECV, %	–	0.850*	0.815*
Native T1, ms	0.850*	–	0.669*
LGE extent, %	0.815*	0.669*	–

*: P < 0.05; Values are Spearman’s rho correlation coefficient; abbreviations as in **Tables 1**, **2**.

### Survival and prognosis-related factors

**Figure 3:**
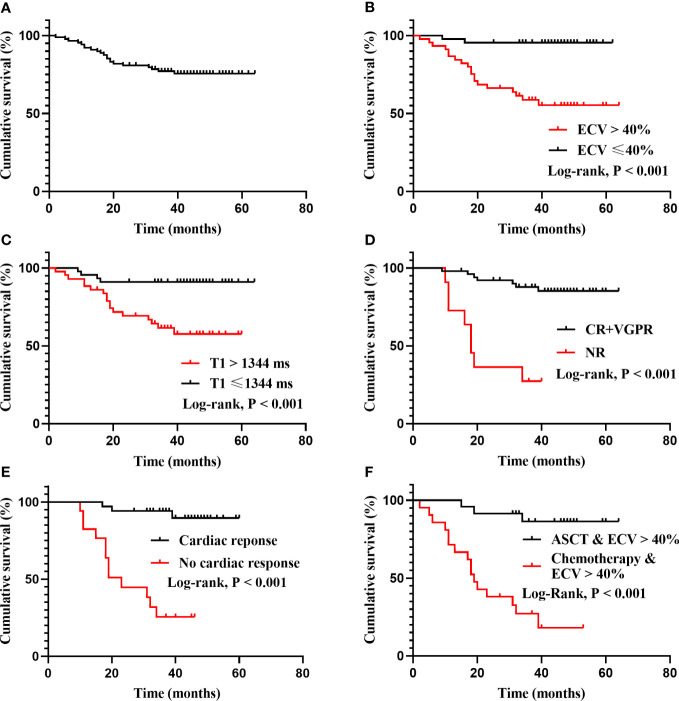
Kaplan-Meier survival curves for total cohort, ECV, native T1, treatment-related responses and treatment regimens. **(A)**: The OS of all patients. The overall 1-, 3- and 5-year estimated OS rate were 92.1%, 77.2% and 75.6%, respectively. The median OS has not been reached. **(B)**: Patients categorized by median ECV (40%) differed significantly in survival probability (95.5% *vs* 55.3% at the fifth year, log rank P < 0.001). **(C)**: Patients with native T1 ≤ 1344 ms and native T1 > 1344 ms differed significantly in survival probability (91.1% *vs* 57.6% at the fifth year, log rank P < 0.001). **(D)**: Patients with deep hematological response (CR+VGPR) and no response (NR) differed significantly in survival probability (log rank P < 0.001). The median OS of the NR group was 18 months. **(E)**: Patients with cardiac response and no cardiac response differed significantly in survival probability (log rank P < 0.001). The median OS of the no cardiac response group was 23 months. **(F)**: In patients with ECV > 40%, ASCT differed significantly in survival probability compared with chemotherapy (log rank P < 0.001). The median OS of chemotherapy group was 19 months.

Patients were followed for a median of 40 months (range, 2-64 months), 21 patients died during follow-up, and 2 patients were lost to follow-up. The median ECV value of deaths was 55.7% (range 34.9% to 74.1%). While the median ECV value of patients receiving ASCT was 35.8% (range 26.4% to 63.6%), and only four patients died in this group, whose baseline ECV values were 53.4%, 43.4%, 39.1%, 61.6%, respectively. The Kaplan–Meier curves for overall survival (OS) are displayed in **Figure 3**, and the overall 1-, 3- and 5-year estimated OS rates in this cohort were 92.1%, 77.2% and 75.6%, respectively (**Figure 3A**).

According to the category by median ECV (40%) and native T1 (1344 ms), the survival probability was differed significantly (**Figures 3B, C**). Patients who achieved a deep hematological response (CR+VGPR) or cardiac response had a higher survival probability, while the median OS of the no hematological response group and the no cardiac response group was 18 months and 23 months, respectively (**Figures 3D, E**). In patients with ECV > 40%, ASCT could significantly improve prognosis compared with chemotherapy (P < 0.001), and the median OS for patients receiving chemotherapy was 19 months (**Figure 3F**).

As displayed in **Table 5**, univariate analysis showed that ECV (per 10% increase), native T1 (per 100 ms increase), LGE extent (per 10% increase), LVEF (per 10% increase), LVMI (per 10 g/m^2^ increase), LV GPWT (Diast), NT-proBNP (per 100 pg/ml increase) and Log (TnT) (per unit increase) were significant predictors of mortality. Given that ECV and native T1 had a significant correlation (rs=0.85), they were analyzed in separate multivariate Cox regression models. The final results of multivariate analysis were shown as follows: ECV (per 10% increase) was a significant prognosis factor for mortality (HR: 2.087, 95% CI: 1.379-3.157, P < 0.001) after correcting for NT-proBNP (HR: 1.017 for per 100 pg/ml increase, 95% CI: 1.001-1.034, P=0.043), and the Harrell’s C-index was 0.817 (95% CI: 0.747-0.888). Native T1 (per 100 ms increase) was a significant prognosis factor for mortality (HR: 2.443, 95% CI: 1.381-4.321, P=0.002) in another multivariate Cox model correcting for NT-proBNP (HR: 1.020 for per 100 pg/ml increase, 95% CI: 1.005-1.035, P=0.011), the Harrell’s C-index was 0.804 (95% CI: 0.715-0.892). In short, only ECV and Native T1 were independently prognostic for mortality in this study.

**Table 5:** Univariate and multivariate Cox proportional hazard analysis in all AL patients.

	Univariate		Multivariate model 1		Multivariate model 2	
Variables	HR (95% CI)	P	HR (95% CI)	P	HR (95% CI)	P
Age	1.021 (0.964-1.083)	0.474	–	–	–	–
Systolic blood pressure, per 10 mmHg increase	0.753 (0.560-1.011)	0.059	–	–	–	–
NT-proBNP, per 100 pg/ml increase	1.030 (1.018-1.042)	<0.001	1.020 (1.005-1.035)	0.011	1.017 (1.001-1.034)	0.043
Log (TnT), per unit increase	13.708 (4.036-46.551)	<0.001				
LVEF, per 10% increase	0.553 (0.401-0.762)	< 0.001				
SVI, per 10 ml/m²	0.576 (0.326-1.020)	0.059	–	–	–	–
LV GPWT (Diast), mm	1.284 (1.142-1.444)	<0.001				
LVMI, per 10 g/m^2^ increase	1.375 (1.185-1.596)	<0.001				
Native T1, per 100 ms increase	3.149 (1.895-5.235)	<0.001	2.443 (1.381-4.321)	0.002	–	–
ECV, per 10% increase	2.528 (1.763-3.626)	<0.001	–	–	2.087 (1.379-3.157)	< 0.001
LGE extent, per 10% increase	1.193 (1.037-1.372)	0.014				

ECV and native T1 were put into separate models because of a significant correlation (rs=0.850). Stepwise regression analysis was chosen. Abbreviations as in **Table 1** and **Table 2**.

### A novel risk stratification model compared with the Mayo 2004 stage

**Figure 4:**
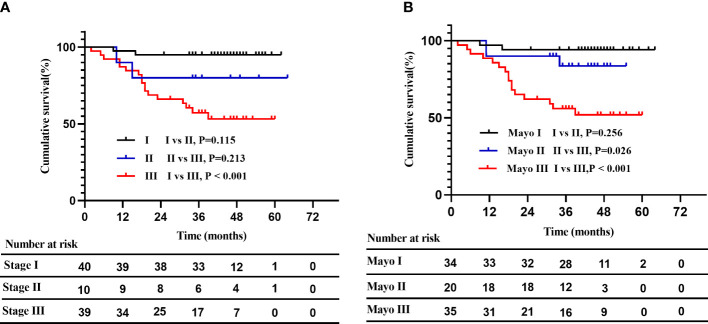
A novel risk stratification model compared with the Mayo 2004 Stage. **(A)**: Kaplan–Meier curves for OS in the novel staging system. The 5-year estimated OS rates in stage I, II, and III were 95%, 80%, and 53%, respectively. Patients with stage I and stage III differed significantly in survival probability (log rank P < 0.001). **(B)**: Kaplan–Meier curves for OS in the Mayo 2004 Stage. The 5-year estimated OS rates in Mayo 2004 Stage I, II, and III were 94%, 84%, and 52%, respectively.

Given that ECV and native T1 remained significantly associated with mortality in two separate multivariate Cox models, we attempted to establish a novel prognostic staging system based on ECV and native T1. The markers and cut-off values in the prognostic staging system were defined as follows: ECV > 40%, native T1 > 1344 ms. Three stages were defined: stage I: no markers above the cut-off, stage II: one marker above the cut-off, and stage III: both markers above the cut-off. As shown in **Figure 4**, Kaplan–Meier curves for OS were similar between the novel staging system and Mayo 2004 Stage in this cohort. The 5-year estimated OS rates in Stage I, II, and III were 95%, 80%, and 53%, respectively. In contrast, the 5-year estimated OS rates in Mayo 2004 Stage I, II, and III were 94%, 84%, and 52%, respectively. There was a significant difference in survival probability between Stage I and III in our proposed staging system (log rank P < 0.001).

## Discussion

In this study, we described the characteristics of CMR parameters and treatment-related responses in an AL amyloidosis cohort. Our study had three major findings. First, native T1 (per 100 ms increase) and ECV (per 10% increase) were independently predictive of mortality. Additionally, a novel risk stratification model based on native T1 and ECV could identify high-risk populations with AL amyloidosis, and was similar to traditional Mayo 2004 Stage. Finally, in patients with ECV > 40%, receiving ASCT had a higher hematological ORR and lower NRR, followed by a better cardiac and renal response than conventional chemotherapy.

ECV reflects the expansion of myocardial interstitial space and can be used to assess amyloid burden (19). Previous studies have demonstrated that ECV is elevated in cardiac amyloidosis (11, 14). We also found that ECV in CA patients was significantly higher than those in non-CA patients, although the diagnosis of CA was clinical rather than pathological. In our research, patients with ECV > 40% had a significantly poorer prognosis than those with ECV ≤ 40%. Therefore, this group is clinically special and needs appropriate treatment to improve outcomes. In the subgroup analysis, we found that patients with ECV > 40% who received ASCT had a tendency to achieve higher hematological ORR, followed by a better cardiac and renal response rate than chemotherapy. Furthermore, we validated that obtaining cardiac response or deep hematological response could significantly improve prognosis in the cohort. In the context of the above results, ASCT may be a better option for improving prognosis in AL patients with ECV > 40% after exclusion of transplant contraindications. With the increase of ECV, patients’ cardiac architecture may be disrupted and cardiac function deteriorates. Therefore, patients with high ECV values may be a relative contraindication for transplantation, and multicenter collaboration is required to determine ECV cut-off values to select patients suitable for transplantation. We hope that ECV will not only provide predictive information but also guide clinical treatment in AL patients.

Martinez-Naharro et al. reported the results of a single-center study of transthyretin amyloidosis, where the correlation between ECV and native T1 differed between groups when grouped by a cut-off of ECV = 40% (R = 0.735 for ECV < 40%, and R = 0.351 for ECV ≥ 40%) (12). However, our research showed different results in AL amyloidosis. We found that the correlation between ECV and native T1 was similar in different subgroups (rs=0.604 for ECV > 40%, and rs=0.538 for ECV ≤ 40%), which means that ECV and native T1 still have good correlations under low levels of infiltration or high amyloid burden. Given the different biological natures of transthyretin amyloidosis and AL amyloidosis, pathogenic free light chains may be toxic to cardiomyocytes and thus cause myocardial edema (20). Native T1 represents a combination of cellular and interstitial signals, unlike ECV, which represents only interstitial dilation (21). In this context, myocardial edema may affect the value of native T1, which can explain why the research results in AL amyloidosis were different from those in transthyretin amyloidosis. Another attractive finding of this research is that ECV had a stronger correlation with Mayo 2012 Stage than native T1 and LGE extent. The Mayo 2012 Stage is associated with the long-term prognosis of patients, as this staging system includes not only cardiac biomarkers but also dFLC representing the underlying clonal disease burden (22, 23). The good correlation between ECV and Mayo 2012 Stage suggests that ECV may also predict long-term survival, which could be incorporated into the prognostic system in the future.

Our study also focused on the prognostic value of CMR parameters in patients with AL amyloidosis using a 3.0 T clinical scanner. Our study found that both ECV and native T1 have prognostic value after correcting for NT-proBNP. However, a study of 82 AL patients by Lin et al. showed that native T1 was not an independent prognostic factor (13). Differences in research methods may account for the different results. In their study, there was no statistical difference in survival probability (P=0.069, using Tarone-Ware test) among patients classified by median native T1 (1456 ms); therefore, native T1 was not included in the univariate and multivariate analysis. It is currently believed that native T1 is greatly affected by the water content of myocardial tissue and therefore increases significantly in the case of myocardial edema (24). Based on the above facts, we have reason to assume that native T1 in AL amyloidosis may be a powerful independent prognostic factor because of its potential to track myocardial edema and amyloid burden in this subtype of amyloidosis. Our study confirmed this hypothesis and demonstrated that native T1 (per 100 ms increase) was a significant prognostic factor for mortality (HR 2.443, 95% CI 1.381-4.321, P=0.002) after correcting for NT-proBNP. In consideration of the impact of ECV and native T1 on the outcomes of patients, we attempted to establish a novel prognostic staging system based on the two CMR parameters. Patients with ECV > 40% and native T1 > 1344 ms had a poor prognosis, the 5-year estimated OS rate was 53%, and the result was similar to Stage III in Mayo 2004 Stage. However, there was only a significant difference in survival probability between Stage I and Stage III in the novel staging system, the small number of Stage II patients [10, (11%)] in the new staging system may be one of the reasons. The prognostic staging system based on ECV and native T1 is only tentatively proposed and needs to be explored in future studies with a larger sample size.

Native T1 is obtained without the use of contrast agents, which means native T1 can be obtained from patients with advanced renal failure, in whom contrast agents are contraindicated. Lack of requirement for contrast is also attractive because of the reduced cost. Although ECV is the best indicator to reflect amyloid burden, as it directly reflects the expansion of interstitium; our study highlights the prognostic function of native T1, and we recommend that AL patients with contrast contraindications perform T1 mapping with native T1 routinely. However, the acquisition of native T1 depends on the magnetic resonance field strength and scan sequence used, so the normal range of native T1 needs to be determined locally.

Our study has three main limitations. First, our study had a limited sample size and was not validated on an external dataset. Second is that a few patients underwent CMR scanning after treatment, which may have confounding effects due to the cardiotoxic influences of chemotherapy agents. The other is that we lack follow-up CMR data on patients to explore the association between treatment efficacy and changes in CMR parameters.

## Conclusion

In summary, our research demonstrated that both ECV and native T1 were independently predictive of mortality in patients with AL amyloidosis. Subjects with ECV > 40% and native T1 > 1344 ms were clinically special with poor prognosis. In terms of treatment, ASCT was effective and significantly improved the prognosis of patients with ECV > 40%.

## Data availability statement

The raw data supporting the conclusions of this article will be made available by the authors, without undue reservation.

## Ethics statement

The studies involving human participants were reviewed and approved by Clinical Trial Ethics Committee of Jinling Hospital. The patients/participants provided their written informed consent to participate in this study.

## Author contributions

FM and CT performed the research and wrote the paper. GR and JG collected the data. LiZ and WX analyzed the data. XZ guided the interpretation of magnetic resonance parameters, and critically review the content of the article. LoZ and XH designed the research study, obtained study funding and provided administrative support. All authors contributed to the article and approved the submitted version.
