# Supplementary material for: The prognostic value of multiparametric cardiac magnetic resonance in patients with systemic light chain amyloidosis
Source: Front Oncol. 2023 May 3;13:1069788. doi: 10.3389/fonc.2023.1069788 (PMC10189062; doi:10.3389/fonc.2023.1069788)
Supplement: Supplementary file 1 [file Table_1.docx]

| Supplementary Table 1. The baseline characteristics of patients receiving ASCT and chemotherapy | | | |
| --- | --- | --- | --- |
|  | ASCT (n=24) | Chemotherapy (n=21) | P value |
| Age, years | 53.5 ± 8.8 | 53.6 ± 8.8 | 0.958 |
| Gender, male / female, n (%) | 12 (50) / 12 (50) | 13 (62) / 8 (38) | 0.317 |
| Systolic blood pressure, mmHg | 113 ± 13 | 114 ± 17 | 0.818 |
| Lambda restricted, n (%) | 23 (96) | 16 (76) | 0.160 |
| Numbers of involved organ, n (%) | 2.0 (2.0-2.0) | 2.0 (2.0-2.0) | 0.449 |
| Urinary proteinuria, g/24h | 5.0 (3.7-9.5) | 2.5 (1.4-8.7) | 0.073 |
| Serum albumin, g/L | 28.7 (26.2-31.2) | 27.9 (22.0-38.1) | 0.916 |
| Serum creatinine, mg/dl | 0.80 (0.66-0.92) | 0.88 (0.70-1.12) | 0.377 |
| dFLC, mg/L | 77.1 (40.4-133.0) | 132.1 (81.1-189.2) | 0.021 |
| iFLC, mg/L | 95.0 (50.0-144.6) | 149.0 (90.1-202.6) | 0.024 |
| BMPCs, % | 3.0 (2.0-5.4) | 4.8 (2.6-7.0) | 0.043 |
| NT-proBNP, pg/ml | 1339 (684-2357) | 3397 (2165-5053) | 0.001 |
| Troponin T, ng/ml | 0.039 (0.024-0.063) | 0.069 (0.039-0.098) | 0.022 |
| Abbreviations as in Table 1. | | | |
